# Co-receptor usage and prediction of v3 genotyping algorithms in hiv-1 subtype b' from paid blood donors experienced anti-retroviral therapy in chinese central province

**DOI:** 10.1186/1743-422X-7-280

**Published:** 2010-10-22

**Authors:** Shuiling Qu, Liying Ma, Lin Yuan, Wesi Xu, Kunxue Hong, Hui Xing, Yang Huang, Xiaoling Yu, Yiming Shao

**Affiliations:** 1 State Key Laboratory for Infectious Disease Control and Prevention, National Center for AIDS/STD Control and Prevention, Chinese Center for Disease Control and Prevention, Beijing 100050, China

## Abstract

**Background:**

This study explored co-receptor usage and prediction of V3 genotyping algorithms in HIV-1 subtype B' from paid blood donors experienced anti-retroviral therapy in Chinese central province in order to design effectively therapeutic regimen.

**Methods:**

HIV-1 strains were isolated in treatment HIV-1 infections and treatment-naïve HIV-1 infections, then co-receptor usage of HIV-1 strains was identified based on Ghost cell lines using flow cytometry. HIV-1 V3 region was amplified and submitted into web-server (WebPSSM and geno2pheno) to predict HIV-1 co-receptor usage. The feasibility of prediction HIV-1 usage with Web-server assay was analyzed by comparing prediction of V3 genotyping algorithms with HIV phenotype assay based on Ghost cell line.

**Results:**

45 HIV-1 strains and 114 HIV-1 strains were isolated from HIV-1 infections exposed anti-retroviral therapy and treatment-naïve, respectively. 41% clinical viruses from ART patients and 18% from treatment-naïve patients used CXCR4 as co-receptor. The net charge in the V3 loop was significantly difference in both groups. The sensitivity and specificity for predicting co-receptor capacity is 54.6% and 90.0% on 11/25 rule, 50.0% and 90% on Web-PSSM_x4r5_, 68.2% and 40.0% on Geno2pheno_[co-receptor]_.

**Conclusion:**

Dual/mixed/X4 co-receptor utilization was higher in ART patients than treatment-naïve patients. It is should paid attention to predicting HIV-1 co-receptor usage based on V3 genotyping algorithms in HIV-1 subtype B' from paid blood donors experienced anti-retroviral therapy in Chinese central province.

## Background

HIV-1 enters a host cell using the CD4 receptor and co-receptors including the CXCR4 and/or CCR5. In general, R5-tropic strains using CCR5 as co-receptor are responsible for the early stage of infection, while mixed or dual-tropic R5/X4 strains using both CXCR4 and CCR5 as co-receptor, and X4 using CXCR4 co-receptor are detected in more advanced disease stages, and are believed to be associated with more rapid CD4 + T cell decline and accelerate disease progression to AIDS[1]. However the X4 viruses usually coexist with R5 viruses in the viral swarm[2]. There are still 50% patients with late stage HIV-1 B infection having only R5 viruses detectable in treatment-naïve HIV-1 patients[3] but not other HIV-1 subtypes[4,5]. The mechanisms that prompt the evolution towards CXCR4 strains from CCR5 strains are not fully understood. Meanwhile, there were different point of views about HIV-1 co-receptor usage after the patients experienced highly active antiretroviral therapy (HAART). After HAART therapy (59 months [6-240 months]), HIV-1 co-receptor usage was fairly stable[6]. But, some drugs are duty to the preferential suppression of CXCR4-special strains of HIV-1[7].

The third variable loop (V3) sequence of HIV envelope is the major domain associated with HIV co-receptor usage[8]. In general, when the amino acids at codons 11 and/or 25 within the V3 loop is positive charged, the HIV strains usually use CXCR4 as co-receptor. Therefore the 11/25 charge rule is a simple genotypic method to be predicted HIV co-receptor usage. Subsequently, several genotyping algorithms based on V3 loop for predicting HIV co-receptor usage have been published, such as neural networks(NN), decision tree support vector machines(SVM)[9], Position Specific Scoring Matrix approach (PSSM)[10]. However, it reports that current V3 genotyping algorithms are inadequate for predicting X4 co-receptor usage in clinical isolates[11].

Since the first co-receptor antagonist----Maraviroc against HIV-1 was approved in the United States in 2007, which blocking HIV-1gp120 from binding to CCR5, thereby preventing HIV-1 into the host cell. It could effectively inhibit CCR5-tropic strain but not CXCR4-tropic strain, and is a promising agent for treatment-experienced patients infected with multidrug-resistant CCR5 strain[12]. It is necessary to know HIV-1 co-receptor usage before Maraviroc is applied to clinical. Therefore, we collected HIV-1 infections experiencing treatment with reverse transcriptase inhibitors, and isolated HIV-1 strains from HIV-1 infections to evaluate the feasibility that predictes HIV-1 co-receptor usage based on V3 genotyping algorithms.

## Results

### Clinical and general characterization of subjects and viral subtype

**Figure 1:**
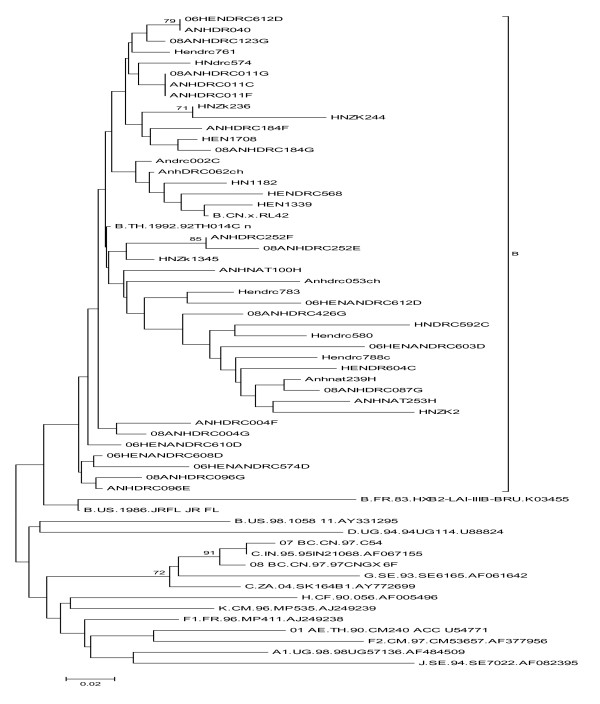
**All the viruses were HIV-1 subtype B' variants (Thai B, a subset of subtype B) through phylogenetic analysis of V3 region gene**. The phylogenetic tree showed that variance of all the HIV viruses are close to B.FR.HXB2 (HIV-1 subtype B) and closer to B.CN.RL42 (Thai B', a clade of HIV-1 B)(see Figure 1).

45 HIV-1 strains were isolated in treatment HIV-1-infection from Anhui (22 strains) and Henan (23 strains) provinces. The mean age was 41 years (26-61 years), 25(60%) of them was women, 17(40%) male. The mean CD4 + T count was 169 (7-901) per μl of whole blood, while the mean plasma viral load (VL) was 4.9(2.7-6.6) log10 HIV-1 RNA copies per ml. The mean treatment time was 26 (6-48 months), of which 23 (51%) patients were from Henan, treatment regimen for the AZT + DDI + NVP; 22 (49%) were from Anhui, treatment regimen for D4T + DDI + NVP (see table 1). All the HIV-1 strains were HIV-1 subtype B' (Thai B, a subset of subtype B) through phylogenetic analysis of V3 region gene. The phylogenetic tree showed that they are close to B.FR.HXB2 (HIV-1 subtype B) and closer to B.CN.RL42 (Thai B', a clade of HIV-1 B) (see Figure 1).

**Table 1:** Characteristics of the participants

	Treatment-naïve group N = 114	ART group N = 45
Sex *n *(%)		
Female	42(36%)	25(60%)
Male	72(64%)	17(40%)
age (years)	43(26-67)	41(26-61)
Plasma HIV-1 RNA level (log10 copies/ml)	4.7 (2.6-7.5)	4.9(2.7-6.6)
CD4^+^T count (cells/μl)	354 (6-917)	169(7-901)
Therapeutic regimen		
AZT+DDI+NVP		22
D4T+DDI+NVP		23
Duration of treatment (months)		26(6-48)
High-risk behavior	Former blood donors	Former blood donors

114 subtype HIV-1 B' strains were isolated in treatment-naïve HIV-1 infections in Anhui province. Their mean age was 43 years (26-67 years old), of which 42(36%) were women, male 72 (64%). The CD4 + T count was 354 (6-917) per μl of whole blood, and the VL was 4.7(2.6-7.5)log10 HIV-1 RNA copies per ml(see table 1). All patients were infected by HIV-1 subtype B' variants through phylogenetic analysis of HIV-1 gene sequence[3,13,14].

### Association of HIV-1 co-receptor usage with clinical characteristics

**Figure 2:**
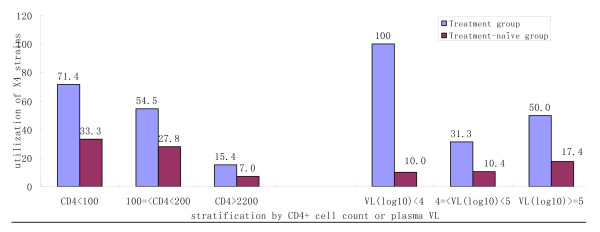
**Association between HIV-1 co-receptor usage and CD4 count or plasma VL**. (A) CXCR4-HIV-1 co-receptor usage utilization decreases with higher CD4 + T cell count in both groups.(B) There is no obviously correlation between VL and HIV-1 co-receptor usage (see Figure 2).

Compared with treatment-naive participants, a higher percentage of HIV-1 strains in treated participants were harboring dual/mixed/X4-tropic viruses (51.1% vs. 18%) (See table 2). To further analyze association of HIV-1 co-receptor usage with clinical characteristics, CD4 + T cell count or VL was stratified and the discrepancy was analyzed using the Mantel-Haenszel test. After adjusted by CD4 + T cell count or VL, the HIV-1 co-receptor usage was difference between treatment-naïve and ART group(p < 0.05; see table 2). HIV-1 X4 co-receptor usage utilization has higher percentage in ART group than treatment-naïve group, and increased with CD4 + T cell count decrease and with VL increase (Figure 2).

**Table 2:** HIV-1 co-receptor usage and its associated influence factors

	Treatment-naïve group N = 114		ART group N = 45		
	**R5 co-receptor usage utilization**	***X4 co-receptor usage *utilization**	**R5co-receptor usage utilization**	***X4 co-receptor usage *utilization**	

CD4+T count (cells/μl)					
CD4 < 100	8(66.7%)	4 (33.3%)	6(28.6%)	15(71.4%)	p = 0.007
100 = < CD4 < 200	13(72.2%)	5 (27.8%)	5(45.5%)	6 (54.5%)	
CD4 > = 200	78(92.9%)	6(7.1%)	9(84.6%)	2 (15.4%)	
VL					
VL(log10) < 4	18(90.0%)	2(10.0%)	0 11(68.7%)	7(100.0%)	
4 = < VL(log10) = < 5	43(89.6%)	5(10.4%)	11(50.0%)	5(31.3%)	p < 0.0001
VL(log10) > 5	40(82.6%)	6(17.4%)		11(50.0%)	
Therapeutic regimen					
AZT+DDI+NVP			9(40.9%)	13 (59.1%)	p = 0.30
D4T+DDI+NVP			13(56.5%)	10 (43.5%)	
Treatment time (months)					
< 18			5(55.6%)	4 (44.4%)	P = 0.88
18-30			10(45.5%)	12 (54.5%)	
>= 30			7(50.0%)	7 (50.0%)	

Note: p is used to test the difference of X4 distribution between treatment-naïve and ART group.

In treatment groups, there is no association between HIV-1 co-receptor usage and therapeutic regimens (p > 0.05). Also, when treatment time was stratified (treatment time < 18 months as a group, 18 months = < treatment time < 30 months as second group, and treatment > = 30 months as the third group), there was no evidence for association between treatment time and HIV-1 co-receptor usage (P > 0.05)(see table 2).

### Association of HIV-1 co-receptor usage with V3 loop sequence

81 sequences of V3 region from the 114 treatment-naïve patients and 42 sequence of V3 region from 45 ART patients were amplified. According to the formula (V3 net charge = (R + K)-(D + E)), net charge of V3 loop was calculated. In the formula, the R and K was short for argentine and lysine, respectively; D and E short for aspartic acid and glutamic acid, respectively. In the ART group, the net charge of V3 loop was distributed from 2 to 7(4.33 ± 1.34), of which 4.86 ± 1.25 for X4/R5 strain, 3.75 ± 1.21 for the R5 strain. In the treatment-naïve group, net charge of V3 was distributed from 2 to 7(4.02 ± 1.02), of which 4.53 ± 0.74 for X4/R5 strain, 3.91 ± 1.05 for R5 strain (see table 3).

**Table 3:** Association of HIV-1 co-receptor usage with the net charge of V3 loop

Characteristic				**Distribution **^**b **^**and frequency **^**c **^**of net charge of V3 loop**					
**Groups**	**tropism**	**N**^**a**^	**Mean ± Std**	**2(%)**	**3(%)**	**4(%)**	**5(%)**	**6(%)**	**7(%)**

Drug-naïve	R5	66	3.91 ± 1.05	**3(3.7)**	**22(27.2)**	**25(37.9)**	12(18.2)	2(3.0)	2(3.0)
	X4/R5	15	4.53 ± 0.74	0		***9(60.0)***	***4(26. 7)***	***2(13.3)***	0
ART	R5	20	3.75 ± 1.21	**1(5.0)**	**7(35.0)**	**7(35.0)**	3(15.0)	2(10.0)	0
	X4/R5	22	4.86 ± 1.25	1(4.6)	3(13.6)	5(22.7)	***4(18.2)***	***7(31.8)***	***2(9.1)***

Note: a: the number of cases;

b:it is the net charge of V3 loop according to the formula (V3 net charge=(R+K)-(D+E));

c:it is the frequency of b in the a.

**bold**: No. of net charge of V3 for R5-tropic viruses distributed mainly below 4, occupied more than 70%;

***bold and italic: ***No. of net charge of V3 for X4-tropic viruses distributed mainly above 4 in drug-naïve group, above 5 in ART group, occupied more than 60%

In both ART and treatment-naïve group, number of net charge of V3 for R5-tropic viruses distributed mainly below 4, which frequency is more than 70%. However, number of net charge of V3 for X4-tropic viruses distributed mainly above 4 in treatment-naïve group, above 5 in ART group (see table 3).

HIV-1 co-receptor usage was predicted based on genotypic algorithm including 11/25 charge rule, Webserver(Web-PSSM_x4r5 _and Geno2pheno_[coreceptor]_), which is called HIV-1 co-receptor genotype. The consistency between genotype and phenotype of co-receptor usage was evaluated among ART population. The sensitivity and specificity for predicting X4 capacity is 54.6% and 90.0% on 11/25 rule, 50.0% and 90% on Web-PSSM_x4r5_, 68.2% and 40.0% on Geno2pheno_[coreceptor]_(see table 4).

**Table 4:** HIV-1 co-receptor prediction based on genotypic algorithm and its sensitivity and specificity in ART population

Methods	prediction HIV-1 co-receptor usage		Consistency with phenotypic	
	
	CCR5 (%)	CXCR4 (%)	sensitivity	specificity
11/25 rule	28(65.1)	15(34.8)	54.6%	90.0%
WebPSSM	29(67.4)	14(32.6)	50.0%	90.0%
geno2pheno	15(34.8)	28(65.1)	68.2%	40%

## Discussion

In 1993, HIV-1 infection of paid blood donors in the central Chinese province of Henan and Anhui provinces constitutes a major epidemic in China[15]. In September 2003, the "Four Frees and One Care" policy was implemented, which provided free antiretroviral drugs in above areas[16]. In this article, we report that a large-scale study of HIV-1 co-receptor usage and their prediction based on V3 genotyping algorithms in population who were infected by paid blood donors in Henan and Anhui province. However, there is limited information to know X4-to-R5 switch of HIV-1 in this population after antiviral therapy. Therefore, the present study was based on the characterization of specimens collected from 45 subjects experienced ART and 114 treatment-naïve subjects between 2005 and 2008. All the viruses isolated from ART and treatment-naïve population in this study are HIV-1 B' subtype. The combination of B' viral subtype and Chinese host's genetic background has likely provided a unique situation for the understanding of HIV-1 co-receptor usage and their prediction based on V3 genotyping algorithms in a particular population who infected though paid blood donation and then experienced ART.

In present study, co-receptor usage of HIV-1 in patients with and without treatment on HAART was detected based on Ghost cell lines (phenotypic assays). The results showed that the HIV-1 CXCR4 utilization among antiretroviral therapy HIV-1 infected patients was higher than in the treatment-naïve population, implying that it should pay attention to the choice of co-receptor antagonists after the treatment failure on HAART. The present study was in agreement with Hunt's results that there is more widely X4-tropism strain in antiretroviral-experienced patients[17]. When CD4 + T cell count or VL was stratified, the HIV-1 co-receptor usage was difference between treatment-naïve and ART group. HIV-1 CXCR4 utilization has higher percentage in ART group than treat-naïve group, and increased with CD4 + T cell count decrease and with VL increase in both group. Usually, the CXCR4 utilization is higher in more advanced disease stages. There is a report that some drugs are duty to the preferential suppression of CXCR4-special strains of HIV-1[7], However, the frequency of CXCR4 utilization in the two therapeutic regimens (AZT + DDI + NVP or D4T + DDI + NVP) is no difference in our study, This study could not found any association between treatment time with CXCR4 utilization, which agreed with other report[6]. HIV-1 R5 to X4 switch is dynamic processes during the interaction between HIV-1 variation and host immune. Of course, it does not exclude the reason that the criterion that the participants in ART group would initiate antiretroviral therapy is that their CD4 + T counts must be blow 200 cells/μl in China.

Number of net charge of V3 plays an important role in detecting viral R5-to X4 co-receptor switch. 70% R5-tropic viral net charge of V3 distributed below 4 whatever exposed to drug or not. However, there is more than 60% for X4-tropic viruses which number of net charge of V3 distributes mainly above 4 in treatment-naïve group, above 5 in ART group, For exception, there is not any X4-tropic viruses which the number of net charge of V3 is below 4 in treatment-naïve group, whereas there is 18.2% X4-tropic viruses which the number of net charge of V3 is below 4 in ART group, suggesting the number of net charge of V3 is not available for co-receptor prediction of HIV-1 B' subtype exposed to drug.

V3 loop, as the major determinant of viral tropism, is a base of lots of prediction essays of co-receptor usage, for example, networks(NN), decision tree[9], support vector machines(SVM) [9], Position Specific Scoring Matrix approach (PSSM)[10]. In this study, PSSM_x4/r5_, geno2pheno_[coreceptor] _and 11/25 charge rule were chosen to assess the concordance with phenotype assay. The specificities and sensitivities in our study is lower than Garrido's study that the specificities for detecting HIV-1 B X4 variants are 92%(PSSM_x4/r5_), 88%(geno2pheno_[coreceptor]_), and the sensitivities are 90%(PSSM_x4/r5_) and 90% (geno2pheno_[coreceptor]_)[18], but higher than Whitcomb's study that the specificities for detecting HIV-1 B X4 variants are more than 90%(PSSM_x4/r5_, geno2pheno_[coreceptor]_, 11/25rule). And the sensitivities are merely 30.5%(11/25rule), 24.5%(PSSM_x4/r5_) and 44.7%(geno2pheno_[coreceptor]_)[11]. The reason for this difference is different method for detecting HIV-1 co-receptor phenotype. Anyway, all the studys reach an agreement that current V3 genotyping algorithms are inadequate for predicting X4 co-receptor usage in clinical isolates.

## Conclusions

In summary, the study shows that prevalence of dual/mixed/X4 HIV-1 strain among ART participants is higher than among treatment-naïve participants. V3 genotyping algorithms for predicting HIV-1 co-receptor usage is not enough for HIV-1 B' subtype from patients experienced ART.

## Methods

### Study population

All the subjects were recruited from HIV-1 infected former blood/plasma donors (FBDs)[13] in the central China. The population with experienced antiretroviral therapies were pre-selected HIV-1-infected patients, who participated in a multicenter AIDS Cohort Study in Anhui and Henan provinces of China during 2005-2008. While the HIV-1 infections without treatment was selected from Anhui province, who were recruited as cohort study of CIPRA (Comprehensive International Program of Research on AIDS) in 2005-2007.

The blood from all the subjects was collected for viral load, CD4 + T count detection and the peripheral blood mononuclear cells (PBMCs) for isolating primary HIV strains. All subjects signed informed consent forms before blood collection. This study was approved by the Institutional Research Ethics Committee of Chinese Center for Disease Control and Prevention in China. The viral load were tested with COBAS AMPLICOR™ techniques and Analyzer (Roche Diagnostics, Alameda, CA). The count of CD4 + T and CD8 + T lymphocytes was performed by flow cytometry (EPICS-XL, Coulter) with TruCount package from BD Biosciences (San Jose, CA).

### HIV-1 isolation from patients' PBMCs

Primary HIV-1 strains were isolated by co-culturing PBMCs from infected individual and those from two or more from healthy individuals after phytohaemagglutinin(PHA)-stimulation. The co-culture was incubated in growth RPMI-1640 medium supplemented with 10% fetal calf serum (FCS), 100 U/ml penicillin, 100 μg/ml streptomycin, 2.9 mg/ml L-glutamine and 100 IU recombinant IL-2 (Roche Diagnostic,Sigma) as previously described[19]. Cultures were maintained by regular addition of uninfected stimulated PBMCs and fresh media. Culture supernatants were collected once a week to measure p24 production levels using a commercial enzyme-linked immunosorbent assay (ELISA) kit according to the instructions from the manufacturer (BioMerieux, Marcy-l'Etoile, France). Virus culture supernatants with p24 consentations higher than 2 ng/ml were aliquoted and stored in liquid nitrogen until being used.

### Detection of HIV-1 co-receptor usage

GHOST cells, expressing CD4 while expressing CXCR4 or CCR5, were seeded in 24-well plates (Corning Inc,Spain) at the density of 1×105 cells/well*0.5 ml. On the following day, the monolayers, about 70% confluent, were infected with virus stocks (200 μl/well) in the presence of 8 μg/ml DEAE-dxtran to enhance the infective efficiency. After 48 hours, cells were harvested and analyzed with flow cytometer (Elite ESP, Beckman Coulter, Germany) and a total of 10,000 to 15,000 events were scored. We expected an approximately 10 fold shift in mean GFP fluorescence of infected cells over uninfected cell[20]. The Ghost-R5 and -X4 cells infected with HIV-1_SF33_, HIV-1 _Ba-L _and HIV-1_IIIB _were positive controls and the cells without HIV-1 infection were negative control.

### Amplification for HIV-1 V3 loop

RNA was extracted from HIV isolates using a RNA Mini Kit (QIAGEN, Germany). Nested polymerase chain reaction was used to sequence the V3 region using the external primers 44F/35R(5'-ACAGTRCARTGYACACATGG-3'/5'-CACTTCTCCAATTGTCCITCA-3), and the internal primers 33F/48R(5'-CTGTTIAATGGCAGICTAGC-3'/5'-RATGGGAGGRGYATACAT-3'). The responsive and cycling parameters were set according to the Takara Ex Taq PCR kit's specification. The PCR products were purified (Gel Extraction Kit, QIAGEN, USA) and then were done for sequencing on an ABI 377 Sequencer (Applied Biosciences) and analyzed sequence using Mega soft[21].

### Bioinformatic prediction

After alignment, sequences with positively charged amino acids at codons 11 and/or 25 within the V3 loop were classified as having an 11/25 genotype. Then the HIV-1 strain with 11/25 genotype was believed as CXCR4 or CXCR4/CCR5 strain.

Based on HIV-1 V3 loop sequence, HIV-1 co-receptor usage were analyzed using published genotypic algorithm such as PSSMX4/R5 http://indra.mullins.microbiol.washington.edu/webpssm/[22], and geno2pheno[coreceptor] http://coreceptor.bioinf.mpi-inf.mpg.de/[23].

### Statistical analysis

In this study, age, CD4 + T count and treatment time was indication as mean or median and range, and virus load was transformed to log10. The age, CD4 + T count or VL difference between ART and treatment-naïve were performed by using T test, and the distribution of gender between two groups was performed by using chi square test. All the statistical analysis was done on SPSS software (V13.0), and a P value less than 0.05 was considered statistically significant.

## Competing interests

The authors declare that they have no competing interests.

## Authors' contributions

SQ and LY performed the experiment, analyzed the data and draft the manuscript. JH, HX,YL, XY, JS,YH, SQ, YF, LL,SL collected samples and performed the experiments LM and YS designed, supervised and directed the studies. All authors read and approved the final manuscript.

## References

[B1] Brumme ZL Goodrich J Mayer HB Brumme CJ Henrick BM Wynhoven B Asselin JJ Cheung PK Hogg RS Montaner JS Harrigan PR Molecular and clinical epidemiology of CXCR4-using HIV-1 in a large population of antiretroviral-naive individuals J Infect Dis 2005 192 3 466 74 10.1086/431519 15995960

[B2] Connor RI Sheridan KE Ceradini D Choe S Landau NR Change in coreceptor use correlates with disease progression in HIV-1--infected individuals J Exp Med 1997 185 4 621 8 10.1084/jem.185.4.621 9034141 PMC2196142

[B3] Guo YF Ma LY Yuan L Wang SH Sun JP Xu WS Xu JQ Xing H Ong KX Zhang XY Ruan YH Zhang YX Shao YM R5 to X4 coreceptor switch of human immunodeficiency virus type 1 B' and B'/C recombinant subtype isolates in China Chin Med J (Engl) 2007 120 6 522 5 17439749

[B4] Ndung'u T Sepako E McLane MF Chand F Bedi K Gaseitsiwe S Doualla-Bell F Peter T Thior I Moyo SM Gilbert PB Novitsky VA Essex M HIV-1 subtype C in vitro growth and coreceptor utilization Virology 2006 347 2 247 60 10.1016/j.virol.2005.11.047 16406460

[B5] Huang W Eshleman SH Toma J Fransen S Stawiski E Paxinos EE Whitcomb JM Young AM Donnell D Mmiro F Musoke P Guay LA Jackson JB Parkin NT Petropoulos CJ Coreceptor tropism in human immunodeficiency virus type 1 subtype D: high prevalence of CXCR4 tropism and heterogeneous composition of viral populations J Virol 2007 81 15 7885 93 10.1128/JVI.00218-07 17507467 PMC1951291

[B6] Lehmann C Daumer M Boussaad I Sing T Beerenwinkel N Lengauer T Schmeisser N Wyen C Fatkenheuer G Kaiser R Stable coreceptor usage of HIV in patients with ongoing treatment failure on HAART J Clin Virol 2006 37 4 300 4 10.1016/j.jcv.2006.08.008 17005445

[B7] Philpott S Weiser B Anastos K Kitchen CM Robison E Meyer WA Sacks HS Mathur-Wagh U Brunner C Burger H Preferential suppression of CXCR4-specific strains of HIV-1 by antiviral therapy J Clin Invest 2001 107 4 431 8 10.1172/JCI11526 11181642 PMC199259

[B8] Pollakis G Kang S Kliphuis A Chalaby MI Goudsmit J Paxton WA N-linked glycosylation of the HIV type-1 gp120 envelope glycoprotein as a major determinant of CCR5 and CXCR4 coreceptor utilization J Biol Chem 2001 276 16 13433 41 10.1074/jbc.M009779200 11278567

[B9] Pillai S Good B Richman D Corbeil J A new perspective on V3 phenotype prediction AIDS Res Hum Retroviruses 2003 19 2 145 9 10.1089/088922203762688658 12643277

[B10] Jensen MA van 't Wout AB Predicting HIV-1 coreceptor usage with sequence analysis AIDS Rev 2003 5 2 104 12 12876899

[B11] Low AJ Dong W Chan D Sing T Swanstrom R Jensen M Pillai S Good B Harrigan PR Current V3 genotyping algorithms are inadequate for predicting X4 co-receptor usage in clinical isolates AIDS 2007 21 14 F17 24 10.1097/QAD.0b013e3282ef81ea 17721088

[B12] Lieberman-Blum SS Fung HB Bandres JC Maraviroc: a CCR5-receptor antagonist for the treatment of HIV-1 infection Clin Ther 2008 30 7 1228 50 10.1016/S0149-2918(08)80048-3 18691983

[B13] Xu JQ Wang JJ Han LF Xu C Ruan YH Xu ZH Chen X Liu ZD Wang J Su B Ding XP Gao B Gu YB Cao XY Xing H Hong KX Peng H Zhao QB Yuan L Feng Y Zhang GY Ma LY Wu L Shao YM Epidemiology, clinical and laboratory characteristics of currently alive HIV-1 infected former blood donors naive to antiretroviral therapy in Anhui Province, China Chin Med J (Engl) 2006 119 23 1941 8 17199937

[B14] Jian-jun WS-hXHW Sequence analysis of gag and env genes of HIV type 1 circulating in former blood donors of Fuyang city, Anhui province National medical journal of china 2007 87 22 17785105

[B15] Wu Z Liu Z Detels R HIV-1 infection in commercial plasma donors in China Lancet 1995 346 8966 61 2 10.1016/S0140-6736(95)92698-4 7603178

[B16] He N Detels R The HIV epidemic in China: history, response, and challenge Cell Res 2005 15 11-12 825 32 10.1038/sj.cr.7290354 16354555

[B17] Hunt PW Harrigan PR Huang W Bates M Williamson DW McCune JM Price RW Spudich SS Lampiris H Hoh R Leigler T Martin JN Deeks SG Prevalence of CXCR4 tropism among antiretroviral-treated HIV-1-infected patients with detectable viremia J Infect Dis 2006 194 7 926 30 10.1086/507312 16960780

[B18] Garrido C Roulet V Chueca N Poveda E Aguilera A Skrabal K Zahonero N Carlos S Garcia F Faudon JL Soriano V de Mendoza C Evaluation of eight different bioinformatics tools to predict viral tropism in different human immunodeficiency virus type 1 subtypes J Clin Microbiol 2008 46 3 887 91 10.1128/JCM.01611-07 18199789 PMC2268339

[B19] Ma L Guo Y Yuan L Huang Y Sun J Qu S Yu X Meng Z He X Jiang S Shao Y Phenotypic and genotypic characterization of human immunodeficiency virus type 1 CRF07_BC strains circulating in the Xinjiang Province of China Retrovirology 2009 6 45 10.1186/1742-4690-6-45 19442296 PMC2693499

[B20] Vodros D Tscherning-Casper C Navea L Schols D De Clercq E Fenyo EM Quantitative evaluation of HIV-1 coreceptor use in the GHOST3 cell assay Virology 2001 291 1 1 11 10.1006/viro.2001.1163 11878871

[B21] Kumar S Nei M Dudley J Tamura K MEGA: a biologist-centric software for evolutionary analysis of DNA and protein sequences Brief Bioinform 2008 9 4 299 306 10.1093/bib/bbn017 18417537 PMC2562624

[B22] Jensen MA Li FS van 't Wout AB Nickle DC Shriner D He HX McLaughlin S Shankarappa R Margolick JB Mullins JI Improved coreceptor usage prediction and genotypic monitoring of R5-to-X4 transition by motif analysis of human immunodeficiency virus type 1 env V3 loop sequences J Virol 2003 77 24 13376 88 10.1128/JVI.77.24.13376-13388.2003 14645592 PMC296044

[B23] Sing T Low AJ Beerenwinkel N Sander O Cheung PK Domingues FS Buch J Daumer M Kaiser R Lengauer T Harrigan PR Predicting HIV coreceptor usage on the basis of genetic and clinical covariates Antivir Ther 2007 12 7 1097 106 18018768

